# Enabling genomic island prediction and comparison in multiple genomes to investigate bacterial evolution and outbreaks

**DOI:** 10.1099/mgen.0.000818

**Published:** 2022-05-18

**Authors:** Claire Bertelli, Kristen L. Gray, Nolan Woods, Adrian C. Lim, Keith E. Tilley, Geoffrey L. Winsor, Gemma R. Hoad, Ata Roudgar, Adam Spencer, James Peltier, Derek Warren, Amogelang R. Raphenya, Andrew G. McArthur, Fiona S. L. Brinkman

**Affiliations:** ^1^​ Department of Molecular Biology and Biochemistry, Simon Fraser University, Burnaby, BC, Canada; ^2^​ Institute of Microbiology, Lausanne University Hospital and University of Lausanne, 1011 Lausanne, Switzerland; ^3^​ Research Computing Group, Simon Fraser University, Burnaby, BC, Canada; ^4^​ David Braley Centre for Antibiotic Discovery, McMaster University, Hamilton, ON, Canada; ^5^​ Michael G. DeGroote Institute for Infectious Disease Research, McMaster University, Hamilton, ON, Canada; ^6^​ Department of Biochemistry and Biomedical Sciences, McMaster University, Hamilton, ON, Canada

**Keywords:** antimicrobial resistance, comparative genomics, genomic islands, interactive visualization, web server

## Abstract

Outbreaks of virulent and/or drug-resistant bacteria have a significant impact on human health and major economic consequences. Genomic islands (GIs; defined as clusters of genes of probable horizontal origin) are of high interest because they disproportionately encode virulence factors, some antimicrobial-resistance (AMR) genes, and other adaptations of medical or environmental interest. While microbial genome sequencing has become rapid and inexpensive, current computational methods for GI analysis are not amenable for rapid, accurate, user-friendly and scalable comparative analysis of sets of related genomes. To help fill this gap, we have developed IslandCompare, an open-source computational pipeline for GI prediction and comparison across several to hundreds of bacterial genomes. A dynamic and interactive visualization strategy displays a bacterial core-genome phylogeny, with bacterial genomes linearly displayed at the phylogenetic tree leaves. Genomes are overlaid with GI predictions and AMR determinants from the Comprehensive Antibiotic Resistance Database (CARD), and regions of similarity between the genomes are also displayed. GI predictions are performed using Sigi-HMM and IslandPath-DIMOB, the two most precise GI prediction tools based on nucleotide composition biases, as well as a novel blast-based consistency step to improve cross-genome prediction consistency. GIs across genomes sharing sequence similarity are grouped into clusters, further aiding comparative analysis and visualization of acquisition and loss of mobile GIs in specific sub-clades. IslandCompare is an open-source software that is containerized for local use, plus available via a user-friendly, web-based interface to allow direct use by bioinformaticians, biologists and clinicians (at https://islandcompare.ca).

## Data Summary

1. All code used in the implementation of IslandCompare can be accessed from GitHub at https://github.com/brinkmanlab/IslandCompare.
2. The web interface can be accessed from https://islandcompare.ca.
3. Reference genomes used for processing of draft genomes are from MicrobeDB [1].
4. Genomes used for the blast consistency testing and development and clustering analysis are from Freschi *et al*. (2018) [2] and Hingston *et al.* (2017) [3].
5. Genomes used to generate Fig. 2 were downloaded from pseudomonas.com [4].

**Impact Statement**

Public-health microbiology is increasingly adopting a population-based approach to genomic epidemiology and characterization of bacterial outbreak strains, analysing many closely related isolates together instead of individual genomes. In this context, there is a need to rapidly compare the mobile genetic elements of bacterial genomes, particularly genomic islands (GIs), which are known to disproportionately encode genes involved in virulence and resistance to some antimicrobial drug classes. IslandCompare is a new web-based software application with a user-friendly interface that addresses this need by providing a platform for the prediction, clustering and visualization of GIs and associated antimicrobial-resistance genes across sets of microbial genomes.

## Introduction

The acquisition of foreign genetic material from other microbial genomes, phages or environmental DNA is a major driver of bacterial and archaeal genome evolution [5, 6]. Clusters of genes of probable horizontal origin, commonly termed genomic islands (GIs), often provide adaptive traits that present a selective advantage and can eventually become fixed in the population. GIs disproportionately encode medically important adaptations, including virulence genes [7] and certain antimicrobial-resistance (AMR) determinants [8, 9]. Due to their highly dynamic nature, GIs and plasmids can represent one of the major sources of variation between strains, as was recently described for outbreak and non-outbreak strains of atypical enteropathogenic *Escherichia coli* [10] and for the *Pseudomonas aeruginosa* Liverpool epidemic strain (LES) [11]. Comparative GI analysis is becoming increasingly important as genomic epidemiology becomes a key investigative tool for pathogen outbreak analysis and characterization of microbial gene mobility. With the rapid decrease of sequencing costs and the increasing availability of dedicated databases and tools, whole-genome sequencing is progressively being implemented worldwide as a routine tool for outbreak analysis [12–14]. Furthermore, evolutionary analyses have moved towards larger-scale datasets, requiring adapted tools to track the integration and loss of larger genomic regions that may confer adaptive capabilities to their hosts.

Over the past decade, many algorithms have been developed to predict and visualize GIs, mainly in single genomes, by identifying hallmarks of GIs such as biased nucleotide composition, mobility genes, phage-related genes or direct repeats [15, 16]. However, most GI predictors are released as command-line tools, hampering their use by biologists, and only a few offer standalone graphical user interfaces or web services, often with limited data visualization [16]. IslandViewer was the first tool combining several GI predictors and offering an interactive and integrative data visualization with AMR genes and virulence factors [17–19]. As a result, it has rapidly become one of the most widely used and cited tools for GI prediction. However, IslandViewer does not allow comparative analysis, beyond side-by-side circular plots for a user-submitted and a reference genome.

To facilitate the comparative analysis and visualization of GIs, we have developed IslandCompare, a novel open-source user-friendly web service. IslandCompare offers GI prediction by two of the most accurate predictors, a novel blast-based module to improve cross-genome prediction consistency, GI clustering by sequence similarity and contextualized visualization with a phylogenetic tree for a few to hundreds of microbial genomes. It should aid investigations of bacterial and archaeal genome evolution for larger-scale datasets that are becoming more routinely obtained, including for investigations of pathogen outbreaks.

## Theory and Implementation

### IslandCompare workflow

**Fig. 1.**
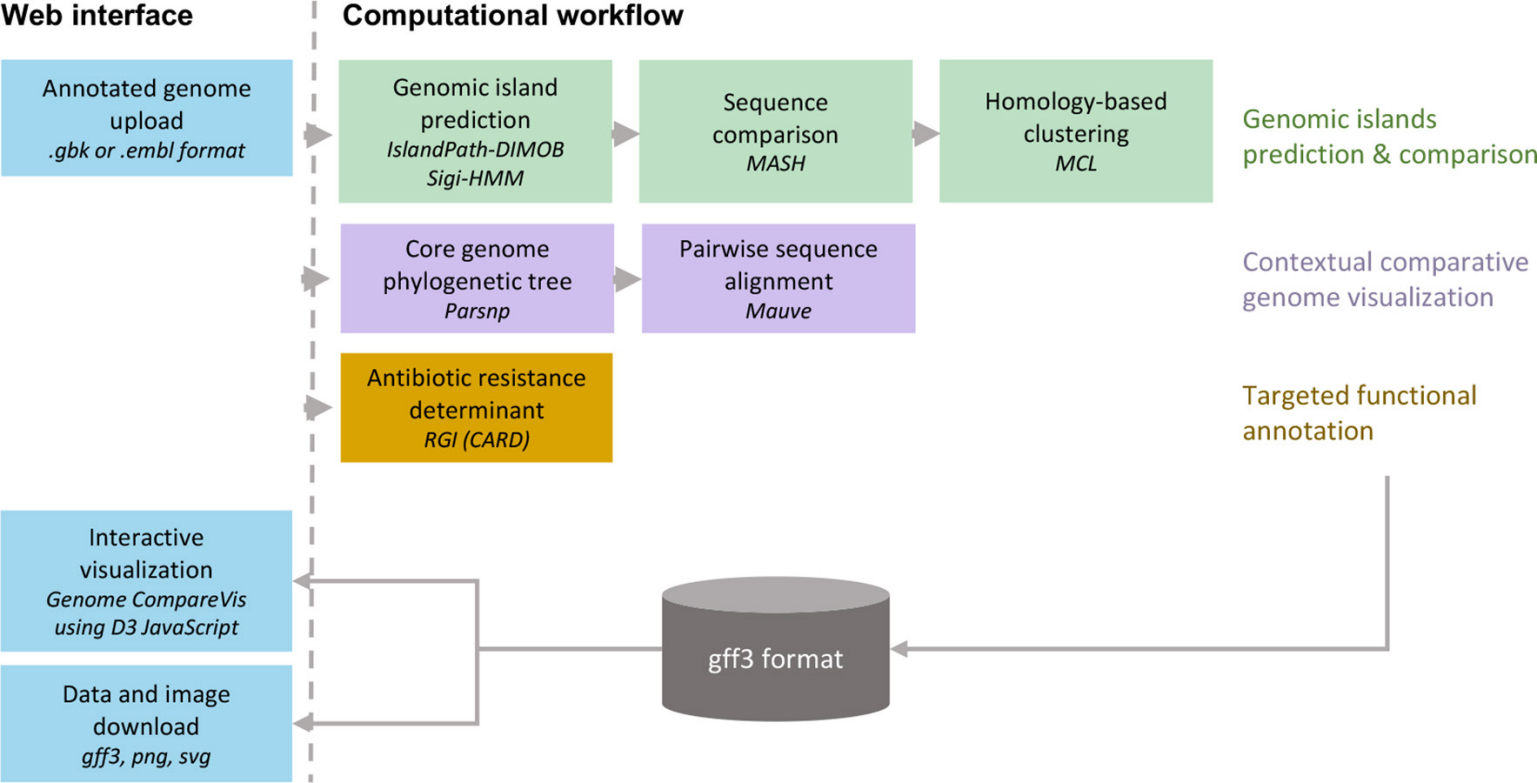
The IslandCompare workflow. IslandCompare integrates three parallel workflows for the prediction and comparison of GIs, the phyletic visualization of genomes, and the annotation and highlighting of genes with potentially interesting functions such as AMR genes. All results are stored in a standard gff3 format that is used either for interactive visualization in the IslandCompare user-friendly web interface, with images available for download, or for the export of data to conduct further GI analyses.

To obtain a comparative view of GIs across several to hundreds of genomes, the IslandCompare workflow includes three parallel pipelines for (i) GI prediction and comparison, (ii) comparative genome visualization, and (iii) identification of AMR determinants (Fig. 1). The analysis workflow is hosted in Galaxy [20] and data processing is supported by a host of integrated tools [21–27]. IslandCompare takes as input GenBank (.gbk) or EMBL (.embl) files of draft or complete genomes with gene and protein annotations. IslandCompare supports draft genome submissions by allowing users to select an existing complete genome to be used as a reference for contig reordering. Contigs that could not be reordered by similarity to the reference genome, including repetitive sequences, will be placed towards the end of the pseudochromosome.

GIs are predicted using two of the most accurate tools available according to our software benchmarking in 2018 [16]: IslandPath-DIMOB [28] and Sigi-HMM [29]. Both tools rely on the identification of sequence composition biases (dinucleotides and codon usage, respectively) in coding regions and, hence, require that genome sequences submitted to IslandCompare are annotated. An additional blast-based consistency step was added to ensure prediction consistency across genomes in an analysis (discussed in greater depth in the following section). To visualize groups of GIs that are similar across genomes, GIs within 500 bp of one another are merged and considered as a single prediction and these GIs are clustered by sequence similarity. Mash [30] applies MinHash, reducing all GI sequences to representative sketches of *k*-mers for comparison, and produces a distance matrix estimated using Jaccard index. This matrix is converted to a weighted graph that is resolved into clusters of similar sequences by the Markov cluster algorithm (MCL) [31].

To fully appreciate potential loss and acquisition of novel GIs, genome visualization must be contextualized with the phylogenetic relationships between the isolates of interest. Therefore, a phylogenetic tree is calculated using Parsnp v1.2 [32] based on SNPs in the core genome of all sequences submitted for analysis. Users wishing to use an existing species phylogeny can provide a tree in Newick format, with branch labels matching genome accession. Then, to parallelize and speed up the computations, pairs of genome sequences ordered according to the phylogeny are compared using Mauve v2015_02_13.0 [33], and regions sharing sequence similarity across the pairs of aligned genomes are displayed as grey areas.

AMR determinants are predicted using the Resistance Gene Identifier (RGI) v5.1.1 of the curated Comprehensive Antibiotic Resistance Database (CARD) v3.0.7 [34, 35]. RGI allows the identification of both protein variants due to gene mutations and protein homologues conferring resistance to antibiotics. IslandCompare only considers resistance determinants identified with the ‘perfect’ label, corresponding to a 100 % amino acid identity match to a resistance determinant in the database, and ‘strict’ label that uses curated bitscore detection cut-offs. Loose labelled resistance determinants are not available in IslandCompare given the large number of spurious hits they may yield.

### Web platform and visualization

**Fig. 2.**
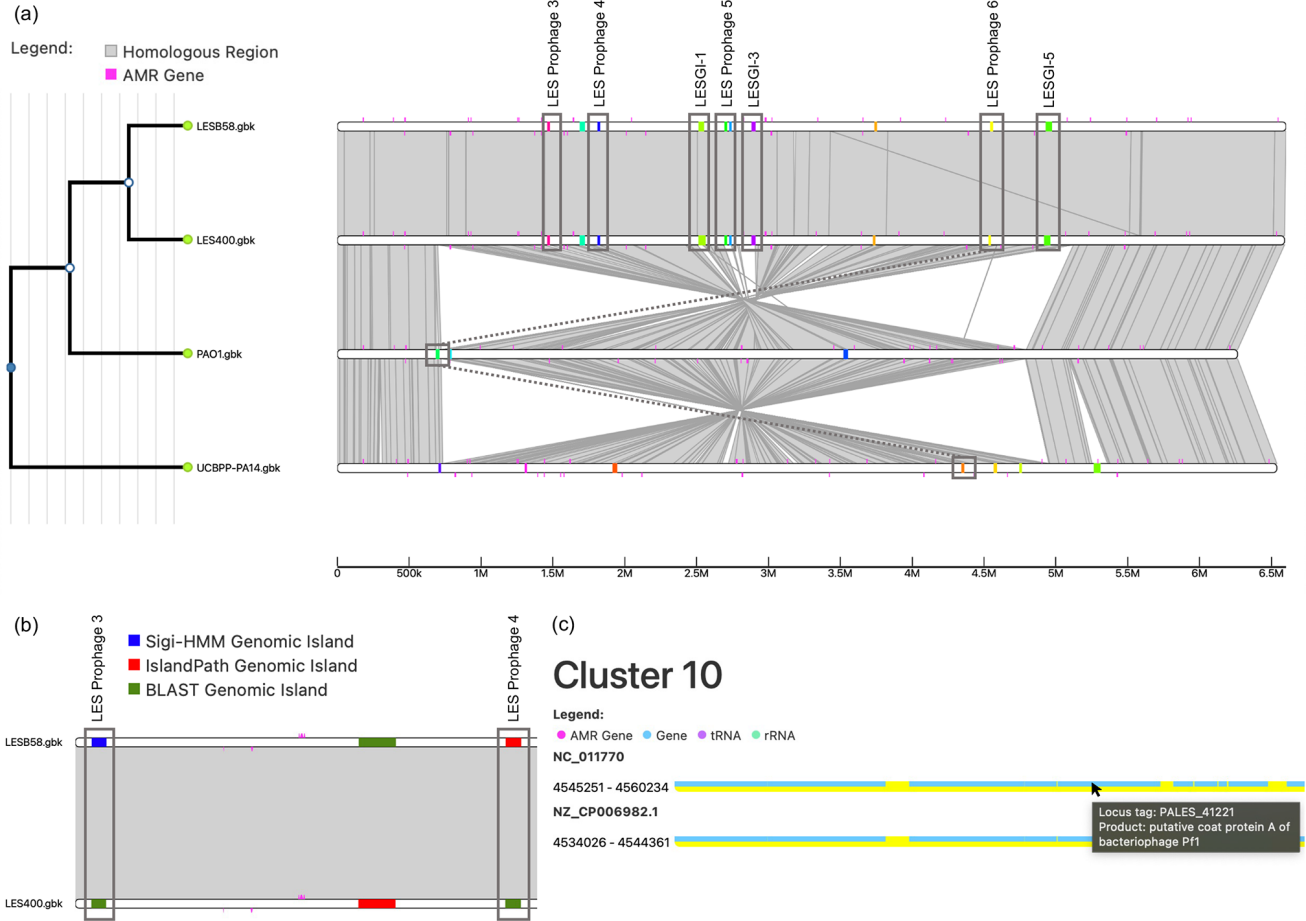
Comparative visualization of four *P. aeruginosa* genomes highlighting GIs and AMR determinants. (a) A phylogeny (left) indicates the relationship between the isolates in the analysis, with zoom-in functionality available. (a – right, b) GIs are represented as coloured blocks placed on a linear representation of the genome (linear white bars indicate genomes, with alignments between genomes shown in grey), with GIs coloured by (a) cluster or (b) prediction method. (**c**) The cluster view allows users to explore gene content within a given GI.

IslandCompare provides a user-friendly web-based interface to allow direct use by bioinformaticians, biologists or clinicians. A drag-and-drop area allows one to easily upload genomes of interest and submit their analysis. A dynamic and interactive visualization strategy displays the bacterial core-genome phylogeny, regions of similarity between genomes, and bacterial genomes overlaid with GI predictions and AMR gene determinants (Fig. 2).

### Data export

To facilitate downstream GI analyses, IslandCompare allows users to download their GI prediction results, as well as the predicted AMR genes and other annotated genome features, in General Feature Format (.GFF). Users can also elect to download only the GI predictions. Images of the phyletic comparative view can be exported to svg and png files for the preparation of publication-grade figures.

### Software availability and web service implementation

IslandCompare version 1.0 can be accessed at https://islandcompare.ca. The web service allows users to submit archaeal and bacterial genomes of interest. Each account has a unique URL that can be bookmarked to access the results at any time during 3 months after data analysis. Older results will be deleted automatically. A separate command line interface tool (https://github.com/brinkmanlab/islandcompare-cli) is available to enable analyses to be submitted and retrieved from the command line, an ideal option for those wishing to process larger numbers of genomes at once. For users wishing to install their own instance of IslandCompare, the source code is freely released under an MIT license (https://github.com/brinkmanlab/IslandCompare). Furthermore, IslandCompare can be deployed with Docker to a cloud computing cluster by following the instructions in the deployment subdirectory.

### Cross-genome GI prediction consistency

The visual nature of IslandCompare has allowed us to identify for the first time cases where GIs are predicted inconsistently across closely related genomes. Fig. S1 (available with the online version of this article) illustrates such a case where a GI is predicted in a subset of genomes in the analysis, but not in others, despite the fact that a nearly identical sequence is present in these genomes as well. In order to evaluate the cause of these inconsistencies, cases of missed GIs were identified in a set of 40 *P *. *aeruginosa* genomes. After exploring a range of length coverage values (Fig. S2a), sequences that aligned with predicted GIs across a minimum of 95 % of the length were retained in the analysis. By evaluating a subset of these cases, we determined that differences in the underlying annotations across genome files were impacting the downstream predictions made by the GI-prediction software. Of particular note, even seemingly trivial differences in a gene being annotated as a pseudogene in one genome and not as such in another genome could lead to inconsistencies in GI annotation. In one such example, outlined in Fig. S3, there are differences in which genes are labelled as pseudogenes, which in turn impacts the dinucleotide measurements in IslandPath, and subsequently the GI predictions. In 20 % of the GIs considered missed by IslandPath, there was no mobility gene predicted by IslandPath in this region due to underlying annotation differences, which would have prevented a GI prediction from being made. Other differences in genes being predicted/not predicted could impact the oligonucleotide bias measures in the region and have downstream effects on GI prediction.

As a result of these findings, a blast-based consistency step is now incorporated into the IslandCompare workflow. The sequences of all GIs predicted by IslandPath-DIMOB and Sigi-HMM are aligned with the genomes in the analysis by nucleotide blast (blastn). blast hits are filtered (length≥400 bp; identity≥90 %; *E* value ≤1.6e−7) and syntenous blast hits are considered as a single alignment. A given region is considered as aligning to a GI if the length of the total alignment is >5 kb and overall coverage to the GI query >95 %. In cases where a region aligns to a predicted island and a GI is not already predicted in this region, the aligned region will be considered a GI prediction and will be labelled as a GI for all output files and the interactive visualization. This module was evaluated on an additional dataset of 166 *Listeria monocytogenes* genomes to verify selected cut-off values.

### Evaluation of GI predictions

A dataset of 86 genomes with known positive and negative GI regions developed by Bertelli *et al*. [16] was used for computing GI prediction performance metrics. As IslandCompare is intended to be used with sets of related genomes, four reference genomes were selected to run with each test genome; reference genomes were randomly selected from a list of available genomes within the same species. A full list of test genomes and associated references used in the analysis can be found in Table S1; all test and reference genomes were downloaded from RefSeq. Given the impact of annotations on downstream GI predictions, GI prediction metrics were computed for the dataset both with National Center for Biotechnology Information (NCBI) annotations (NCBI Annotation Pipeline versions 3.0–4.12) and Prokka (version 1.13). In addition, all metrics were computed for the results both with and without the new blast-based consistency module included. True positives (TP) and false positives (FP) were identified on a per nucleotide basis for nucleotides predicted as being within GIs that overlapped with the positive and negative datasets, respectively. Nucleotides that were not labelled as a GI were categorized as true negatives (TN) or false negatives (FN) if they overlapped with the negative or positive datasets, respectively. Accuracy, recall, precision, F-score and Matthews correlation coefficient (MCC) were calculated according to the following formulas:

$$Accuracy=\frac{TP+TN}{TP+FP+TN+FN}$$

$$Recall=\frac{TP}{TP+FN}$$

$$Precision=\frac{TP}{TP+FP}$$

$$F1\;score=\frac{2TP}{2TP+FP+FN}$$

$$MCC=\frac{TP\times TN+FP\times FN}{\left(TP+FP)(TP+FN)(TN+FP)(TN+FN\right)}$$

### Evaluation of GI clustering method

A range of Mash *k*-mer sizes and cluster granularity parameters for the MCL step were evaluated to ensure optimal clustering. GI predictions and clusters were generated in IslandCompare for datasets of 166 *L *. *monocytogenes* genomes and 40 *P *. *aeruginosa* genomes. blastn [36] was used to determine which GIs aligned to one another. Syntenous blast alignments separated by less than 6 kb were merged and GIs with a total alignment length spanning >50 % of the sequence length were considered as cluster pairs (all cut-offs were determined empirically by assessing a range of values). These cluster pairs were used to determine which GI cluster pairs predicted by IslandCompare were true/false predictions. Accuracy, recall and precision were calculated according to the same formulas used for the GI prediction evaluation.

## Results and Discussion

IslandCompare has been developed to enable direct GI prediction, comparison and explorative visualization across many genomes. The visual output (Fig. 2) features an interactive and linear representation of each genome overlaid with GI predictions. The tree can be displayed as a phylogram or a cladogram, by toggling branches, and a simple click on internal nodes allows the user to select sub-clades of interest for visualization. A zoom-in functionality enables visualization of genes and their annotations for a selected genomic region. GIs are coloured uniformly according to their sequence cluster and hovering over one allows the user to highlight similar GIs across all genomes. Selecting a GI brings in a specific view of all members of the cluster with its respective position in each genome, gene annotations and flagged AMR determinants, which will be expanded with further information in the future.

**Fig. 3.**
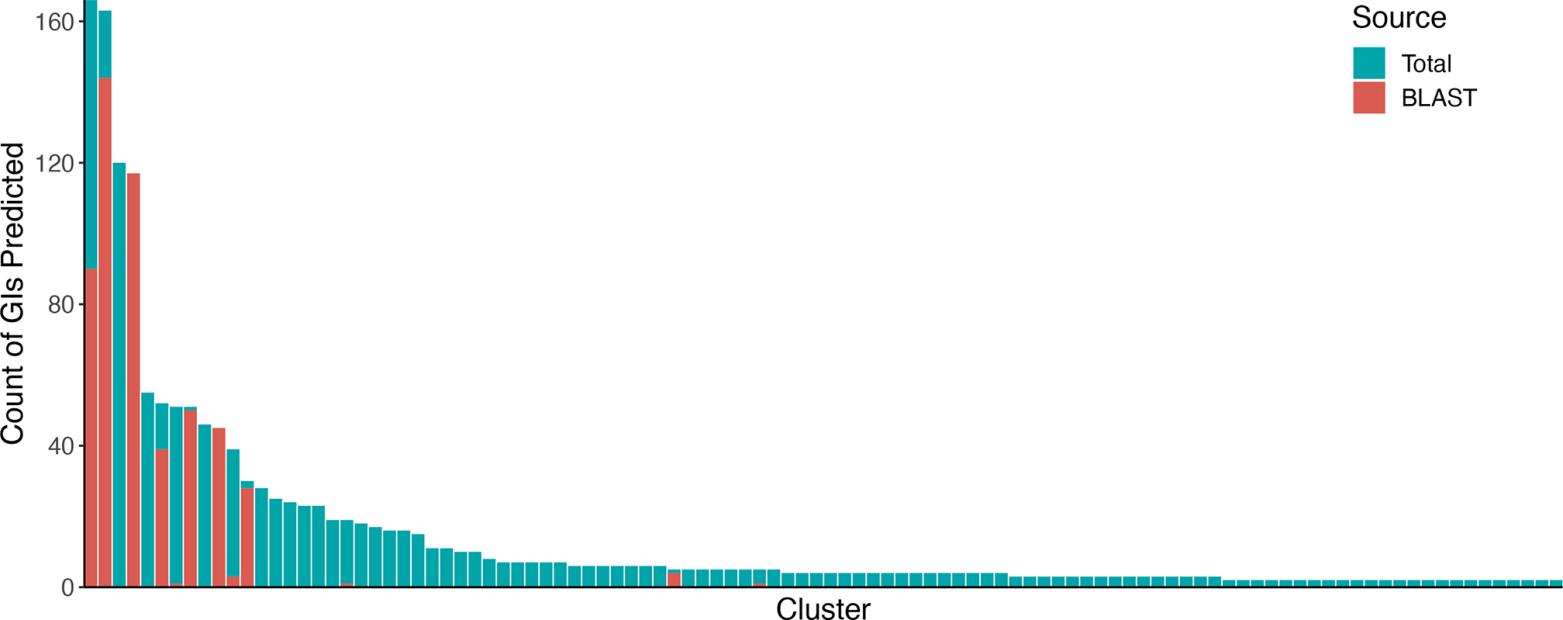
Counts of predicted GIs for each cluster and proportion predicted by the blast-based consistency module for a dataset of 166 *L *. *monocytogenes* genomes. Only clusters with more than one GI sequence predicted in the dataset are represented here (see Fig. S4a, b for all clusters).

An evaluation of the blast-based consistency module, which was integrated to ensure that GIs are predicted consistently across genomes within the analysis, in a dataset of *L. monocytogenes* genomes indicated that the selected length coverage threshold of 95 % is effective at retaining only the spike of nearly identical sequences targeted by this module (Fig. S2b). For this dataset, the blast-based consistency module predictions contributed to 600/1595 merged GI results. The proportion of GI predictions made by blastn within a single cluster were highly variable (Fig. 3), indicating that some GIs were more prone to being missed while others were predicted very consistently by the composition-based GI prediction tools (see Fig. S4a for the proportion of blast-based consistency module predictions by cluster for the *P. aeruginosa* dataset). While the new blast-based consistency module will help improve GI prediction consistency across genomes in IslandCompare, these results allude to the general importance of ensuring annotation consistency for downstream analyses (again, see Fig. S3). Similar issues would be expected to arise for other gene-annotation-dependent analyses, including prediction of AMR genes and microbial typing (depending on the software used).

**Fig. 4.**
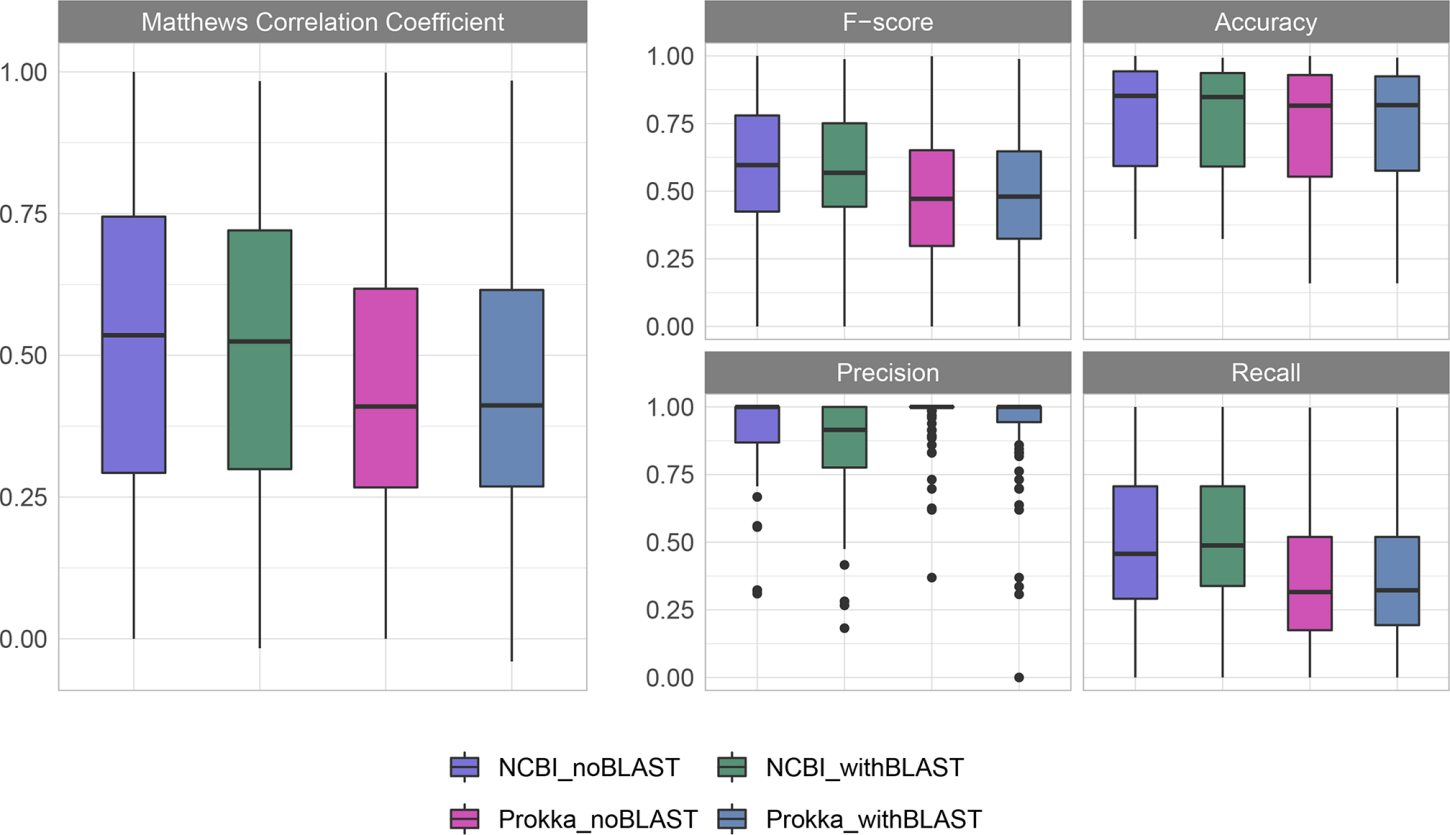
GI prediction metrics across 86 genomes with known positive and negative GI regions. Predictions were made on the same set of genomes annotated by either NCBI or Prokka. All analyses were run for the GI results both with and without the blast-based consistency module results included.

Overall, the GI predictions made on the NCBI-annotated genomes produced slightly higher MCC values and F-scores, although the Prokka-annotated genome predictions had moderately higher precision, and accuracy was comparable across all sets (Fig. 4, Table 1). This analysis was performed with the same genomes annotated by two different platforms, NCBI and Prokka, due to the aforementioned importance of gene annotations in the downstream prediction of GIs. There were more predictions made when the genomes were annotated with NCBI than with Prokka. With a greater number of predictions made, it would be expected that the number of true positives recalled when using NCBI is higher (hence, the higher recall). However, this is at the expense of some additional false positives, impacting the precision. The results with just the IslandPath-DIMOB and Sigi-HMM predictions (noBLAST) were also compared to the complete results with the blast-based consistency module added. Generally speaking, the addition of this module afforded a slight increase in recall at the expense of a small dip in precision, although the difference was moderate. Compared to the previous analysis of IslandPath-DIMOB and Sigi-HMM [16], the MCC and F-score values were higher in the NCBI_noBLAST results (combination of IslandPath-DIMOB and Sigi-HMM than for either individual tool. For all other metrics (accuracy, recall, precision) the results for the NCBI_noBLAST most closely resembled the best result for either IslandPath-DIMOB or Sigi-HMM from this previous study, despite the fact that an intermediate result would have been expected. This could be due to the updated NCBI annotations in all genomes used in the analysis, the slight variation in the dataset (which contains fewer genomes than the previous study due to reference availability), or some combination thereof. The decision of which annotation platform to use and whether to include the blast-based consistency module results will depend upon the application and priorities of a given user. For example, if a user wishes to obtain as many GI predictions as possible and confidently compare GI content across genomes, then it would be advisable for them to annotate with NCBI and include the blast consistency results in their analysis, but another user wishing to explore the high confidence GI contents of their population as a whole with less regard to differences across individual genomes ought to consider annotating with Prokka and disregarding the blast consistency results.

**Table 1.** Mean GI prediction metrics across 86 genomes annotated with either NCBI or Prokka shown with or without the new blast-based consistency module

Predictor	MCC	F-score	Accuracy	Precision	Recall
NCBI_noblast	0.524	0.592	0.771	0.906	0.497
NCBI_withblast	0.514	0.597	0.767	0.849	0.521
Prokka_noblast	0.439	0.466	0.744	0.963	0.355
Prokka_withblast	0.438	0.473	0.746	0.925	0.366

To illustrate the utility of IslandCompare, we performed an analysis of a set of *P. aeruginosa* genomes (Fig. 2a, b, c). This analysis includes the LES B58 strain, whose GI contents were characterized by Winstanley *et al*. [11], as well as another LES and the reference strains PAO1 and PA14, in order to illustrate how IslandCompare can facilitate the identification of unique GIs. A few key GIs are labelled in the figure, as named in the Winstanley *et al*. publication; IslandCompare identified 7/11 GIs discussed in this study, consistent with the focus of GI predictions on precision (islands identified are highly likely to be true islands) at the expense of recall. Most of these GIs (5/7) were only predicted in one of the two LES isolates by the IslandPath-DIMOB and Sigi-HMM modules (subset of these cases shown in Fig. 2b), but were confirmed to be present in both with the blast-based consistency module included. Two GIs inserted in tandem – LES prophage 5 (broken into two predictions in IslandCompare) and LES GI-3 – are easily identifiable with the IslandCompare visual; it can be seen from the Mauve alignment that these islands are unique to the LES isolates. LES prophage 6, a PF1-like phage, can be seen in both LES isolates and the cluster view of this island is shown in Fig. 2(c). Similar Pf1 islands can be seen in PA14 and PAO1 as well, although for this GI only the LES sequences cluster together. LES prophages 3 and 4, as well as GI-1 and GI-5 can also be easily identified as GIs only present in the LES isolates from this figure. This example analysis demonstrates the effectiveness of IslandCompare for rapidly identifying differences in GI content across closely related genomes.

As IslandCompare integrates a complex comparative pipeline building upon existing tools, the time-to-result can range from minutes to several hours and depends on the number of genomes submitted. Datasets of 20, 100 and 1000 draft *Enterococcus faecium* genomes [37, 38] ran in 22 min, 59 min and 14 h, respectively. Therefore, users who plan to regularly run large analyses (eg >200 genomes) are encouraged to set up IslandCompare on a local server using the containerized version that we provide (instructions under the deployment subdirectory of https://github.com/brinkmanlab/IslandCompare). To favour parallelization and decrease computation time, multiple sequence alignments with Mauve that are time-consuming have been replaced by pairwise alignments, while retaining the sensitivity of the tool. The core genome SNP-based reconstruction of a phylogenetic tree using Parsnp is also a time-consuming step. Furthermore, Parsnp requires that closely related genomes are analysed to run successfully and the inclusion of distant genomes may lead to pipeline abortion. Hence, the user can bypass Parsnp by providing a Newick tree as an additional input file, allowing one to then proceed directly with the pairwise Mauve sequence alignment that handles adequately more distant genome alignments.

**Fig. 5.**
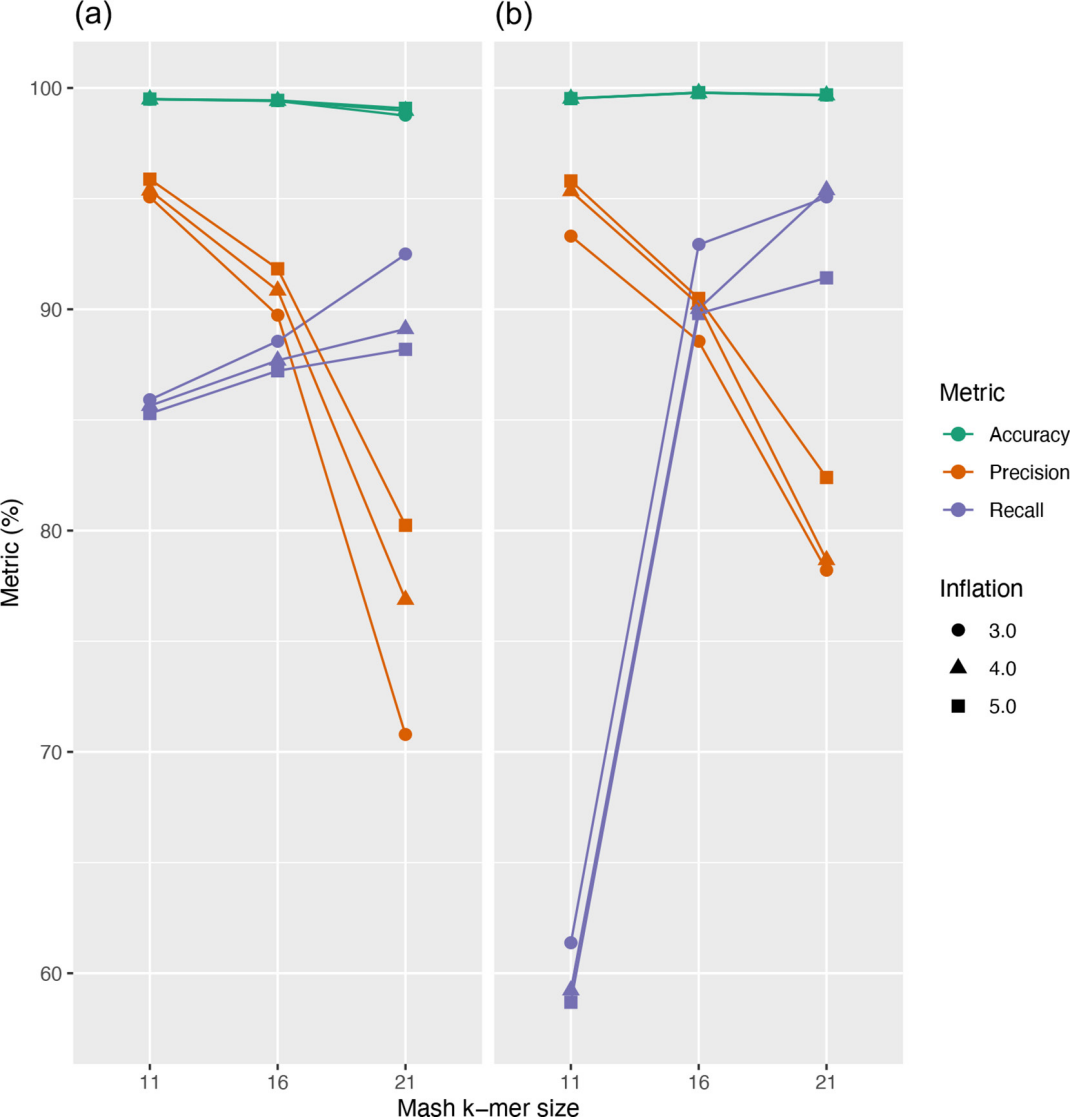
Evaluation of a range of parameters for GI clustering in IslandCompare for datasets of (a) 166 *L *. *monocytogenes* genomes and (b) 40 *P *. *aeruginosa* genomes. Based on this analysis, a *k*-mer size of 16 and inflation value of 5.0 were selected for the Mash and MCL steps in the IslandCompare clustering pipeline, respectively.

The clustering of GIs into groups sharing sequence similarity allows users to rapidly identify similar integrated elements shared across genomes. However, GIs are well known to be highly dynamic regions where rapid evolution by mutation or gene loss is often observed [39, 40]. Multiple GI integration in the same location or further gene integration have also been observed [41–43], leading to further sequence diversity. Hence, GIs may rapidly differ and sequence similarity-based clustering may not perfectly capture the complex evolution of these elements. In this first IslandCompare release, the display of region-wide similarity (grey shaded areas) with high genomic synteny and similarity with GIs belonging to different clusters allows one to identify and visualize these cases, before more advanced strategies can be implemented in future releases. Default clustering parameters for IslandCompare were selected to provide a balance between recall and precision, while prioritizing precision (Fig. 5). Additional work is underway to provide targeted prediction of a curated set of GIs described in previous studies for a selection of target pathogens, with 404 GI sequences and associated metadata already collected.

IslandCompare provides users with a user-friendly interface for comparative GI analysis that does not require advanced command-line bioinformatics skills. It combines well-accepted, widely used GI predictors with a novel blast-based component to improve cross-genome prediction consistency that is not available in any other GI prediction tool. This resource should enable more robust comparison of GIs to gain further insights into pathogen evolution.

## Supplementary Data

Supplementary material 1

Supplementary material 2
