# Supplementary material for: Enabling genomic island prediction and comparison in multiple genomes to investigate bacterial evolution and outbreaks
Source: Microb Genom. 2022 May 18;8(5):mgen000818. doi: 10.1099/mgen.0.000818 (PMC9465072; doi:10.1099/mgen.0.000818)
Supplement: Supplementary material 2 [file mgen-8-818-s001.pdf]

Supplementary Figures and Tables

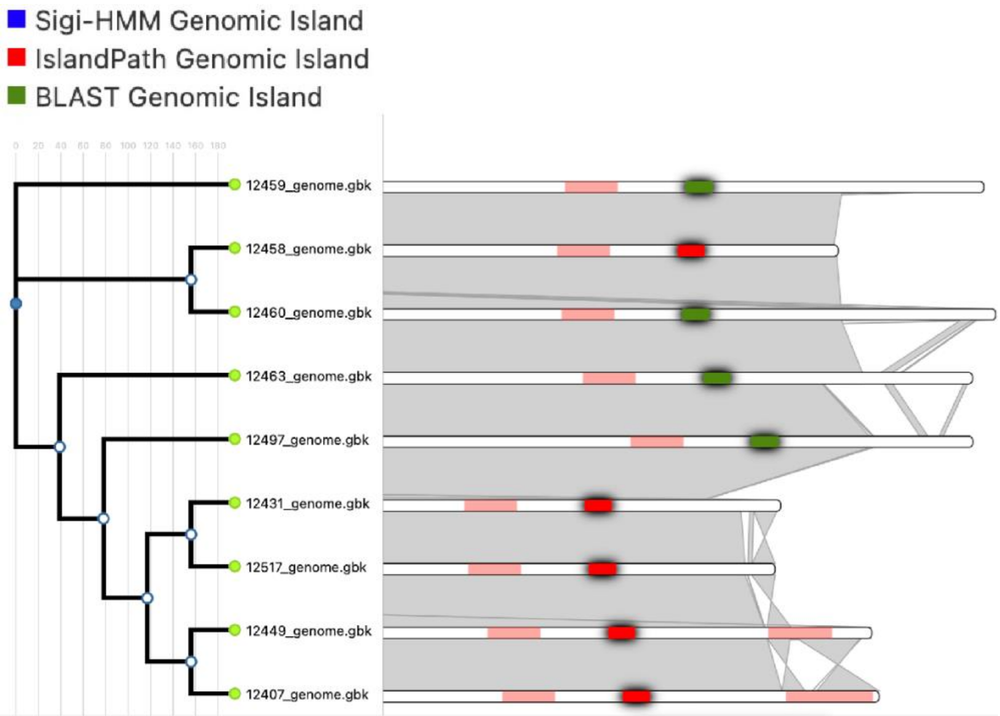

Fig. S1. Output of an IslandCompare run with GIs coloured by prediction method. The highlighted GI was inconsistently predicted by IslandPath-DIMOB (GIs in red) and missed from four of the genomes. The mauve alignment (gray shaded regions) indicates that the predicted GI from adjacent genomes aligns with the corresponding regions. These regions are only predicted as GIs when the BLAST-based consistency module (GIs in green) is included.

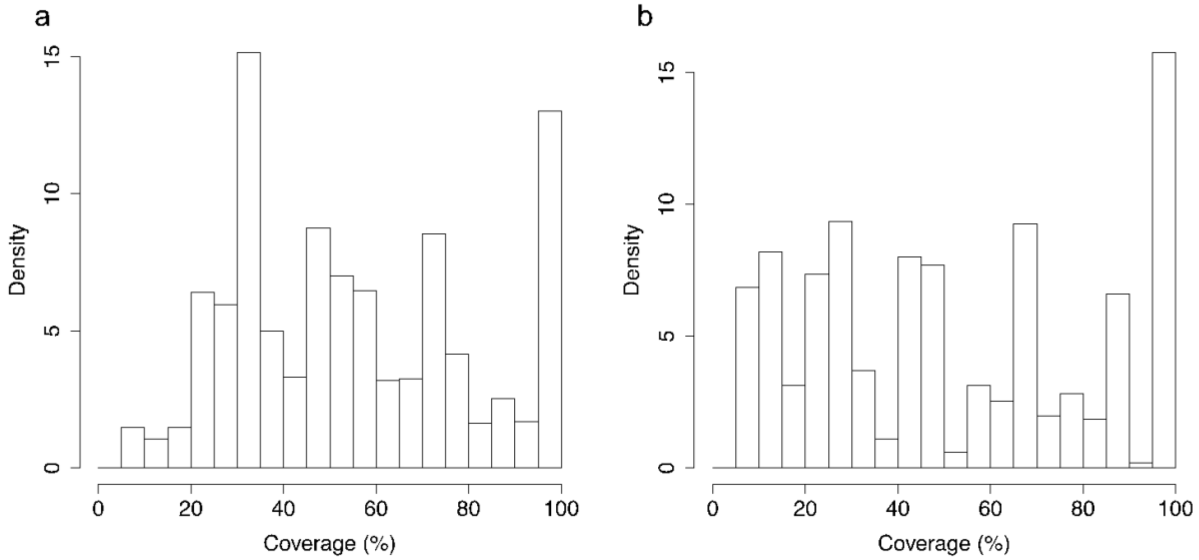

Fig. S2. Distribution of values for the percent coverage of putative missed GIs across the length of their corresponding GI query sequence from the analysis of a) 40 *P. aeruginosa* genomes and b) 166 *L. monocytogenes* genomes.

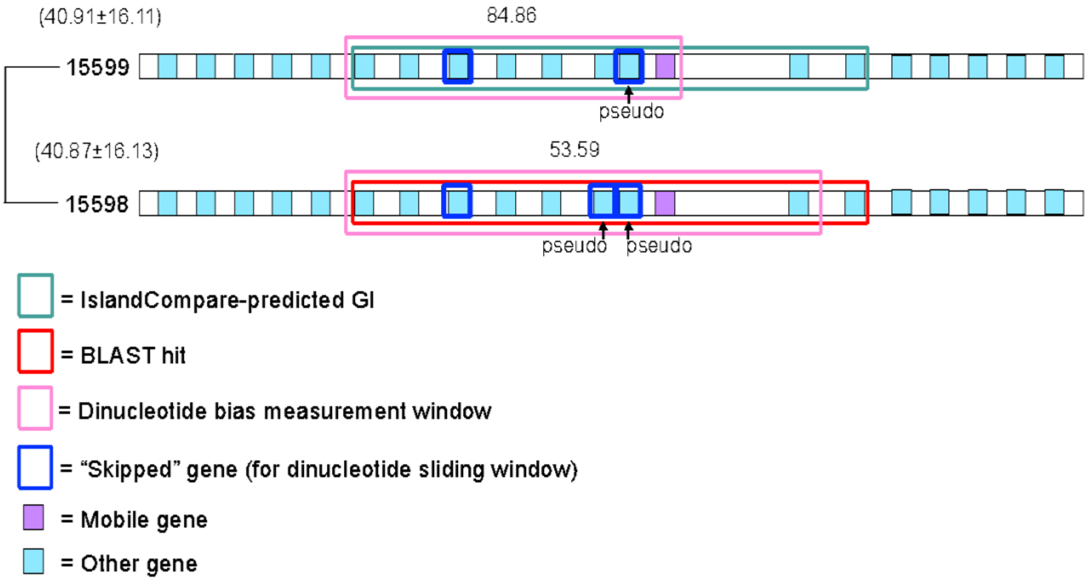

Fig. S3. Impact of alternate pseudogene annotations on the prediction of a GI by IslandPath. A genomic region predicted to be a GI in one genome (top) and an aligned region in a second genome not predicted as a GI (bottom) shown with genes annotated as coloured blocks. Both genomes have a mobility gene (purple) predicted in the putative GI region. One gene is annotated as a pseudogene in only the bottom genome. Due to this annotation difference, the dinucleotide bias measurement windows encompass an alternate set of genes between the two genomes; pseudogenes are not processed by IslandPath. In the top genome, the dinucleotide bias measure of 84.86 exceeds the minimum value to be flagged as a GI (one standard deviation above the median – in this case 40.91+16.11=57.02), while for the bottom genome the measured dinucleotide bias value falls below this cut-off.

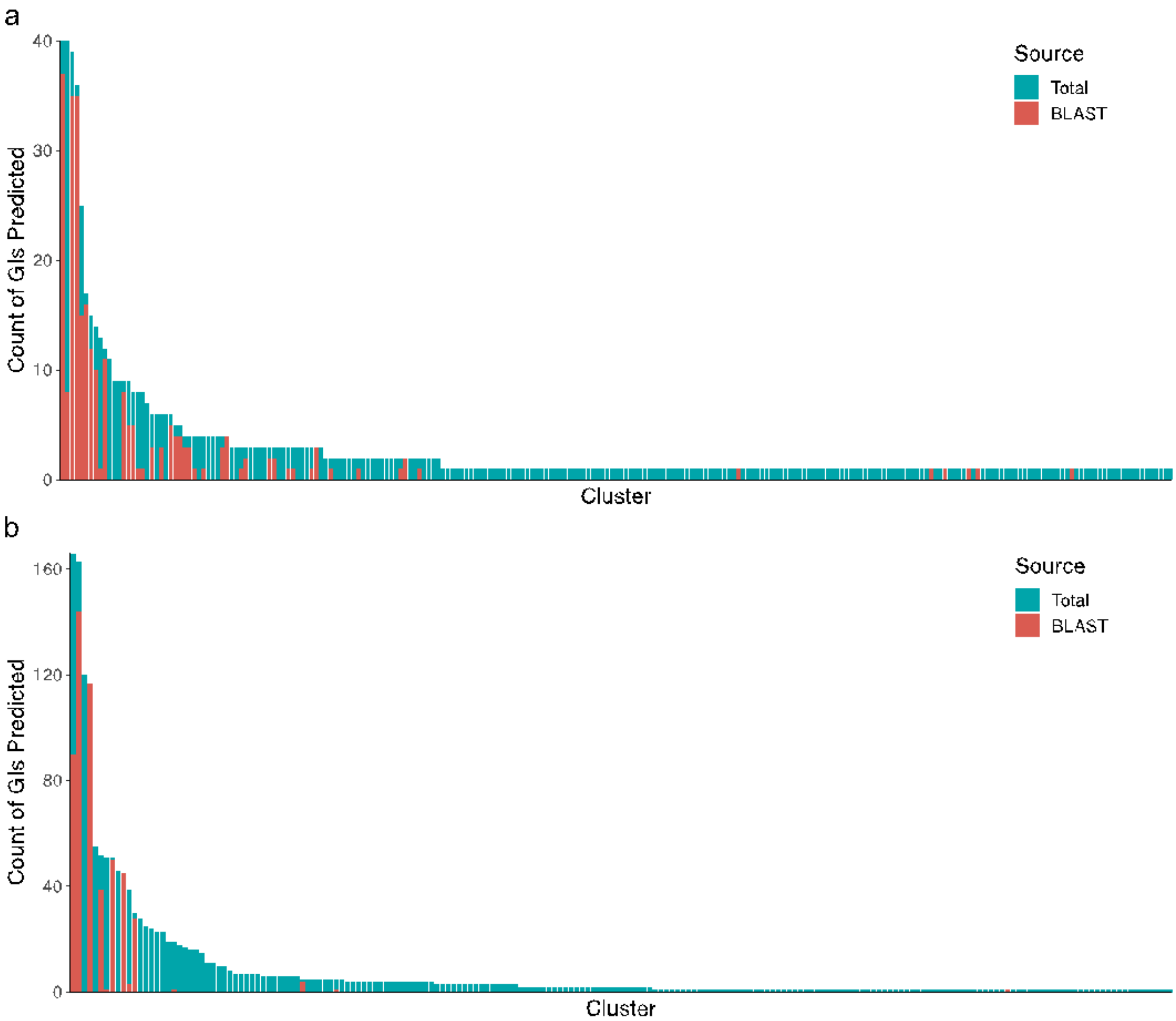

Fig. S4. Counts of predicted GIs for each cluster and proportion predicted by the BLAST-based consistency module for datasets of a) 40 *P. aeruginosa* and b) 166 *L. monocytogenes* genomes.
